# Implications of genetic heterogeneity for plant translocation during ecological restoration

**DOI:** 10.1002/ece3.6978

**Published:** 2021-01-16

**Authors:** Taylor M. Crow, C. Alex Buerkle, Daniel E. Runcie, Kristina M. Hufford

**Affiliations:** ^1^ Department of Plant Sciences University of California Davis CA USA; ^2^ Department of Botany University of Wyoming Laramie WY USA; ^3^ Ecosystem Science and Management University of Wyoming Laramie WY USA

**Keywords:** *Cercocarpus montanus*, ecological restoration, genetic differentiation, genetic structure, niche, phylogeography, seed transfer, seed transfer zones

## Abstract

Ecological restoration often requires translocating plant material from distant sites. Importing suitable plant material is important for successful establishment and persistence. Yet, published guidelines for seed transfer are available for very few species. Accurately predicting how transferred plants will perform requires multiyear and multi‐environment field trials and comprehensive follow‐up work, and is therefore infeasible given the number of species used in restoration programs. Alternative methods to predict the outcomes of seed transfer are valuable for species without published guidelines. In this study, we analyzed the genetic structure of an important shrub used in ecological restoration in the Southern Rocky Mountains called alder‐leaf mountain mahogany (*Cercocarpus montanus*). We sequenced DNA from 1,440 plants in 48 populations across a broad geographic range. We found that genetic heterogeneity among populations reflected the complex climate and topography across which the species is distributed. We identified temperature and precipitation variables that were useful predictors of genetic differentiation and can be used to generate seed transfer recommendations. These results will be valuable for defining management and restoration practices for mountain mahogany.

## Graphical abstract

We analyzed the genetic structure of a common montane shrub that occurs in the Southern Rocky Mountain and is commonly used in restoration projects. We found that environment and topography influenced genetic variation and that genetic diversity was reduced at the environmental margins of its distribution. The results are interpreted to guide seed transfer during ecological restoration.

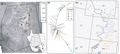

## 1 INTRODUCTION

The restoration of vegetation after a disturbance event can improve ecosystem services (Barral et al., 2015). For example, soil stabilization, pollinator and wildlife habitat, nutrient cycling, and carbon sequestration are all positively correlated with successful ecological restoration (Benayas et al., 2009). However, bringing nonlocal plant material to a restoration site can have unintended consequences. Importing maladapted individuals can result in plant mortality (Johnson et al., 2004), inbreeding depression of introduced material, outbreeding depression within future local and nonlocal hybrid populations (Hufford & Mazer, 2003), or negative biotic interactions (Bucharova et al., 2017). Therefore, importing suitable plant material is important for ecological restoration.

Seed transfer guidelines are intended to establish criteria to aid in the selection of plant material for restoration. However, traditional common garden experiments are expensive and time‐consuming (Johnson et al., 2004), requiring multiyear and multi‐environment field trials and comprehensive follow‐up census work. Typically, the relationship between phenotypic variation and the environmental origin of seed sources are used to create categorical seed transfer zones (Bower & Aitken, 2008; Campbell & Sorensen, 1978), continuous seed transfer guidelines (Parker and Niejenhuis, 1996), or both (Hamann et al., 2000; Saenz‐Romero & Tapia‐Olivares, 2008). These experiments, however, are limited by the number of populations, number of environments, and the amount of time it may take to quantify consequences of importing foreign plant material (Johnson et al., 2004).

Models based on climate (Bower et al., 2014; Crow et al., 2018) or genetic data (Krauss & Koch, 2004), or a combination of both (Massatti et al., 2020) may be useful for establishing seed transfer guidelines without the financial or time investment required by a transplant experiment. For example, the average distance at which significant genetic differentiation is found may be used to guide seed transfer of species (Krauss & He, 2006). Alternatively, multivariate spatial models of environmental tolerance can be used to predict seed transfer (Crow et al., 2018). Genomic analyses are used to identify patterns of genetic variation and structure, which are important for restoration and conservation. For example, gene flow between introduced and native populations may lead to outbreeding depression when locally adapted gene complexes are disrupted by immigrant alleles after admixture (Fenster & Galloway, 2000; Montalvo & Ellstrand, 2001). Identifying geographic and environmental patterns related to genetic differentiation can therefore provide useful guidelines for seed introductions in ecological restoration (Montalvo & Ellstrand, 2001).

Genetic structure of species across space and its association with dimensions of the environmental niche can be used to develop seed transfer guidelines. Although genetic sequence data alone are not a test for the fitness consequences of transferring plant material, it can still be useful for guiding seed transfer. One conceptualization of the species niche is summarized by Hutchison's n‐dimensional hypervolume (Hutchison, 1957), described as a set of biologically relevant and independent environmental axes within which a species occurs. The multivariate environmental space represents conditions that accommodate population persistence and growth (Hutchinson, 1978). As habitat quality or availability decreases and populations become more isolated, genetic variation is expected to decrease (Brown, 1984; Eckert et al., 2008; Sexton et al., 2009). Understanding the relationship between a species' genetic structure and niche can lead to the identification of evolved population differences and locally adapted ecotypes to inform guidelines for seed transfer.

In this study, we investigated genetic structure and variation relevant for restoration of a native perennial shrub, alder‐leaf mountain mahogany, *Cercocarpus montanus*. Mountain mahogany is used in restoration projects because of its value as a forage plant for large ungulates, especially in the winter months (Brotherson, 1992; Turley et al., 2003). We collected and sequenced DNA from 1,440 individual plant samples from 48 populations, estimated genetic diversity within populations, and quantified allelic variation at over 6,000 single nucleotide polymorphisms (SNPs) to describe genetic structure. We tested to what extent genetic structure was a function of climate, geography, topography, or a combination thereof, with the goal of informing seed transfer recommendations for mountain mahogany. We also analyzed the association between genetic variation and climate and geographic range centrality to determine the likely drivers of population demography.

## 2 METHODS

### 2.1 Study species

**FIGURE 1:**
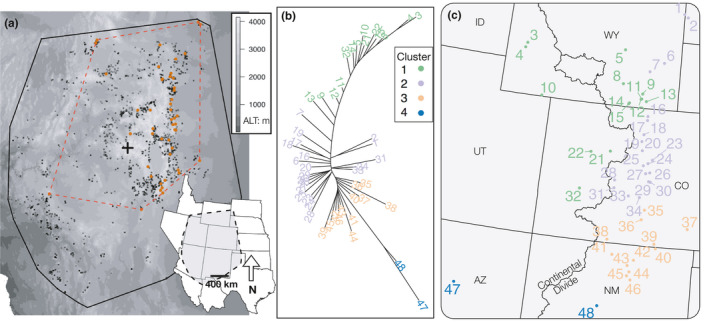
(a) Minimum convex polygon (mcp) of species range (black line) around species occurrence points (black squares), and the dashed red line is a mcp around 48 sampled populations (red diamonds). The geographic center of the overall species distribution mcp is marked with a cross. (b) Unrooted neighbor‐joining tree of Nei's*D*_A_, colors correspond to assigned genetic cluster. (c) Map of sampled populations with numbers from 1 to 48 based on latitude for reference

We selected *Cercocarpus montanus* Raf. because it has a large distribution in the Southern Rocky Mountains and is used in ecological restoration projects (Paschke et al., 2003). Mountain mahogany is a deciduous, perennial shrub species in the rose family (Rosaceae) with a large spatial distribution in western North America (Dorn, 2001). The species occurs on both sides of the Continental Divide and from northern Mexico to the Wyoming–Montana state borders in the United States (Figure 1). Populations are generally distributed between 1,200 and 3,000 m in elevation and often grow in rocky, limestone soils (Williams et al., 2004). Mountain mahogany are monoecious and have wind‐pollinated flowers. Fruits are achenes with an elongated style that twists in later development and are covered in trichomes. These structures are hypothesized to aid in wind‐ and animal‐mediated dispersal (Gucker, 2006). Mountain mahogany shrubs serve as hosts for nitrogen‐fixing actinomycete bacteria (genus *Frankia*) in root nodules, and this adaptation contributes to successional processes in arid regions dominated by unstable, low nitrogen soils (Klemmedson, 1979).

### 2.2 DNA extraction, sequencing, assembly, and variant detection

Mountain mahogany populations were located along a north–south axis in the Southern Rocky Mountains (Figure 1). We collected leaf tissue from 30 individuals in each of 48 populations and extracted DNA using a modified cetyltrimethyl ammonium bromide (CTAB) protocol (Doyle, 1987). DNA was quantified with a NanoDrop 2000 spectrophotometer (Thermo Fisher, Inc.), and additional extractions were conducted when necessary due to high levels of contaminants or low DNA concentrations. We prepared genomic libraries for genotype‐by‐sequencing (GBS) following protocols in Parchman et al. (2012). To summarize, we digested sample DNA with two restriction enzymes (MseI and EcoRI) and ligated barcodes containing unique 8–10 bp sequences to the resulting DNA fragments for each sample to ensure that sequence reads could be assigned to individuals. We then PCR amplified the barcoded restriction–ligation products with standard Illumina primers (1, 5ʹ ‐AATGATACGGCGACCACCGAGATCTACACTCTTTCCCTACACGACGCTCTTCCGATCT ‐3ʹ; 2, 5ʹ ‐CAAGCAGAAGACGGCATACGAGCTCTTCCGATCT‐3ʹ) (Illumina, Inc.).

Barcoded PCR products were combined into two multiplexed libraries of 720 individual samples (with individuals allocated to the libraries randomly to avoid confounding library effects) and sequenced at the University of Texas Genomic Sequencing and Analysis Facility (Austin, Texas, USA) on the Illumina HiSeq 2500 platform using single‐end 100 bp reads. After filtering reads for oligonucleotides used in library synthesis and the PhiX genome, with subsequent demultiplexing and assignment of reads to individuals, we had 24,000,000 sequence reads for further analysis. We completed a de novo genome assembly with a randomly chosen subset of 2.4×10^7^ reads using SEQMAN NGEN software (DNASTAR, Inc.). This step resulted in construction of an artificial, partial reference genome containing 111,967 contigs. We used bwa (Burrows‐Wheeler Aligner; Li & Durbin, 2009) to map reads from each individual to this partial reference genome. Once complete, 15,520,448 total reads (64.6%) assembled to the partial reference genome. Aligned reads were then indexed and sorted using samtools and bcftools (Li et al., 2009). We used the command “mpileup ‐P ILLUMINA ‐u ‐g ‐I ‐f cemo.fasta sorted.bam | bcftools view ‐N ‐c ‐e ‐g ‐v ‐I ‐d 0.8 ‐p 0.01 ‐P full ‐t 0.001 ‐o variants.vcf” to calculate genotype likelihoods and filter variant sites. We then retained a single SNP per contig and removed SNPs with an allele frequency less than 0.05.

### 2.3 Population genetic analyses

Low coverage genotype‐by‐sequencing (GBS) data contain sequencing error. Therefore, we estimated genotypes as the mean of the genotype likelihood distribution and constructed a genetic covariance matrix for all individuals to include the uncertainty inherent in GBS data. We ran a principal components analysis (PCA) of the genetic covariance matrix using the *prcomp* function in *R* to summarize genetic variation.

We used GBS data to estimate ancestral population membership. Genetic cluster algorithms can help explain genetic variation among individuals, and visualize population membership over broad scales (Lawson et al., 2018), such as in our study. Genotype data were used to calculate individual admixture coefficients using the sparse non‐negative matrix factorization algorithm (sNMF) implemented in the LEA package (Frichot & François, 2015; Frichot et al., 2014) in R. This algorithm estimates ancestry coefficients in a computationally efficient manner. The sNMF algorithm is similar to the program STRUCTURE (Falush et al., 2003; Pritchard et al., 2000), which estimates ancestry independently for each individual, and does not require a priori assumptions about population membership. To determine the best supported number of genetic clusters (K) within our collections of mountain mahogany, we used a cross‐entropy criterion from *K* = 1 to *K* = 10 from the snmf function. This criterion uses a masked genotype testing set to determine the prediction accuracy of the model at each *K* value.

The primary aim of this study was to describe the drivers of genetic differentiation among the 48 sampled populations of mountain mahogany (Table S2). We calculated allele frequencies for each of the 6,352 SNPs across the 20–30 sampled individuals per population. Point estimates of allele frequencies within each population were calculated from the genotype likelihoods, and allele frequencies were used to calculate the Weir moment estimator of *F*_ST_ (Weir & Hill, 2002) and Nei's genetic distance (*D*_A_) (Nei et al., 1983; Takezaki & Nei, 1996) as measures of genetic differentiation. *F*_ST_ was calculated using the calculate.all.pairwise.fst function in the BEDASSLE package in R, and *D*_A_ was calculated using a custom R script. We calculated two different genetic differentiation statistics because the two models we used to describe genetic differentiation used different differentiation inputs.

The first model approach we used to describe population genetic differentiation was Bayesian linear models which used Nei's *D*_A_ as the response variable, and pairwise geographic distance, environmental distance, and a binary variable representing the Continental Divide as model predictors. Population pairs were assigned 0 if they originated from the same side of the Continental Divide, or assigned 1 if they were collected from opposite sides of the divide. Environmental distances were measured as the population pair difference for each environmental variable centered on the mean and divided by the standard deviation (z‐score). Climatic variables included thirty‐year normal temperature and precipitation estimates from thin plate spline surfaces (http://forest.moscowfsl.wsu.edu/climate). The precipitation and temperature variable with the highest correlation with Nei's *D*_A_ were selected and combined to serve as the environmental distance predictor in these models. All predictor variables (Table S1) were standardized prior to modeling so that the magnitude of their estimated coefficients could be compared. We fit the models for genetic differentiation in R with the rjags package for MCMC models in JAGS (Plummer, 2003). We ran Markov chain Monte Carlo (MCMC) simulations for 10,000 iterations with the first 2,000 steps discarded as burn‐in. We thinned the MCMC chain every five steps for a total posterior sample of 1,600 for each of three chains. The deviance information criterion (DIC) was used to select the model that best accounted for genetic distance, as well as to compare models with and without spatial distance, environmental distance, and topographic barriers as covariates.

### 2.4 Relative contribution of environment, topography, and geography on genetic differentiation

We used a second type of model specifically designed to quantify the effect of environment relative to geography on genetic differentiation Bradburd et al. (2013). Geographic and environmental distances contribute to adaptive differentiation that can affect translocation outcomes of seed sources. This model was developed by Bradburd et al. (2013), called Bayesian Estimation of Differentiation in Alleles by Spatial Structure and Local Ecology, and is implemented in the R package BEDASSLE. The model is useful for analyzing our data because it uses an MCMC approach that outperforms mantel tests in separating the effects of environment, topography, and geography on genetic differentiation. We tested the complete dataset and used the beta‐binomial Markov chain Monte Carlo model. We ran MCMC simulations for 3 × 10^6^ iterations, thinned the chain every 20 iterations, and checked the trace plots for convergence and acceptance rates.

### 2.5 Population genetic diversity in central and peripheral habitat

The previously described models used genetic differentiation statistics to identify drivers of genetic structure. However, genetic variation within populations is also important to consider for restoration. Specifically, we asked whether variation in population genetic diversity could be explained by geographic or environmental centrality. We estimated genetic diversity for each population using the program ANGSD (Korneliussen et al., 2014). Sequence alignments to the pseudo‐reference (sorted BAM files) were used as input to calculate each population's site allele frequencies (SAF) from genotype likelihoods. We filtered sites that had a minimum mapping quality of 10 and a minimum *q*‐score of 20. The allele frequency likelihoods were used to calculate the maximum‐likelihood estimate (MLE) of the site frequency spectrum (SFS) using the EM algorithm. Estimates of nucleotide polymorphisms were calculated as *θ_π_* (Tajima, 1983), a measure of average pairwise differences, and Watterson *θ_W_* (Watterson, 1975), which is based on the number of segregating sites. Theta estimates were calculated using the empirical Bayesian approach with the SFS as priors (following http://popgen.dk/angsd/index.php/Thetas,Tajima,Neutrality_tests).

If environmental or geographic centrality were associated with genetic diversity, then these metrics could be used to guide seed transfer of mountain mahogany and potentially validate or refute our analysis of genetic differentiation. We used range‐wide occurrence points from a previous study of mountain mahogany to calculate geographic and environmental centrality of each of our 48 populations (Crow et al., 2018). Spatial centrality was calculated as the great circle geographic distance (van Etten, 2018) from each of our sampled populations to the mean latitude and longitude of the species' range (Figure 1). We calculated spatial peripherality as the distance between each population and the shortest linear distance to the edge of the minimum convex polygon of the species' range. Environmental centrality was calculated as the multidimensional Euclidean distance of each population to the species' environmental centroid and the centroid of each genetic cluster (Blonder et al., 2014). Environmental centrality was the summed environmental distance of the top two precipitation and temperature variables that were most correlated with genetic diversity. We also tested the correlation between genetic diversity and the probability of occurrence derived from a previously published species distribution model (SDM) of mountain mahogany (Crow et al., 2018) as an indicator of habitat suitability. We also analyzed whether habitat suitability was correlated with geographic centrality to get a better picture of the niche of this species. In summary, environmental variables were selected for the SDM using a model improvement ratio following (Murphy et al., 2010), and a Random Forest algorithm was used to generate the distribution model.

We used linear models to determine the association between variation in population genetic diversity and environmental and geographic centrality. We used the lm and ANOVA function from the stats packages in R for these models. We also quantified the correlation between the probability of occurrence for the species (taken from a previous study) and geographic range centrality.

### 2.6 Niche similarity among genetic clusters

We used niche overlap statistics to test whether genetic clusters defined by the sNMF admixture analysis occupied distinct subsets of the overall environmental range. Broennimann et al. (2012) developed methods to get an unbiased estimate of niche overlap using kernel smoother functions applied to densities of occurrence points in environmental space, calibrated on the available environmental space across the study area. We calculated kernel densities for the environment occupied by each genetic cluster and used *D* metrics (Schoener, 1970) to determine whether there was significant overlap of niche space between genetic groups:

$$D=1‐0.5\left(\sum_{\mathrm{ij}}\left|z_{1_{\mathrm{ij}}}‐z_{2_{\mathrm{ij}}}\right|\right)$$

where $z_{1_{\mathrm{ij}}}$ and $z_{2_{\mathrm{ij}}}$ are the occupancy of the environment calculated from kernel density functions of entity one and two, respectively. The *D* metric is 0 if there is no overlap between genetic groups and 1 if there is complete overlap. We used the ecospat package (Broennimann et al., 2017) in R (R Core Team, 2018) to calculate niche similarity and overlap. Ecospat performs a randomization test where $z1_{\mathrm{ij}}$ and $z2_{\mathrm{ij}}$ are combined and randomly separated into two groups, and the *D* statistic is calculated 100 times to build a null distribution. The observed *D* statistics, using genetic clusters as entity designations, were calculated and compared with the distribution of simulated *D* values for each pair of genetic clusters separately. Presence points and environmental data for the distribution of mountain mahogany from Crow et al. (2018) were incorporated as background points.

## 3 RESULTS

### 3.1 Sequence alignment and SNP discovery

We identified 12,022 single nucleotide variants using samtools and bcftools (Li & Durbin, 2009). For a variant site to be identified, we required that at least 50% of all individuals have a minimum of one read at that locus. After removing sites with a minor allele frequency of <5% and randomly selecting one variant per contig to ensure independence of loci, we retained 6,352 single nucleotide polymorphisms (SNPs) for further analyses of population genetic structure. In sum, 1,366 of the 1,440 individual samples of *C. montanus* had sufficient sequencing coverage to be retained for further analysis, resulting in a range of 22–30 individuals per population. Remaining samples each had an average of 8.5 reads per SNP.

### 3.2 Population genetic analyses

**FIGURE 2:**
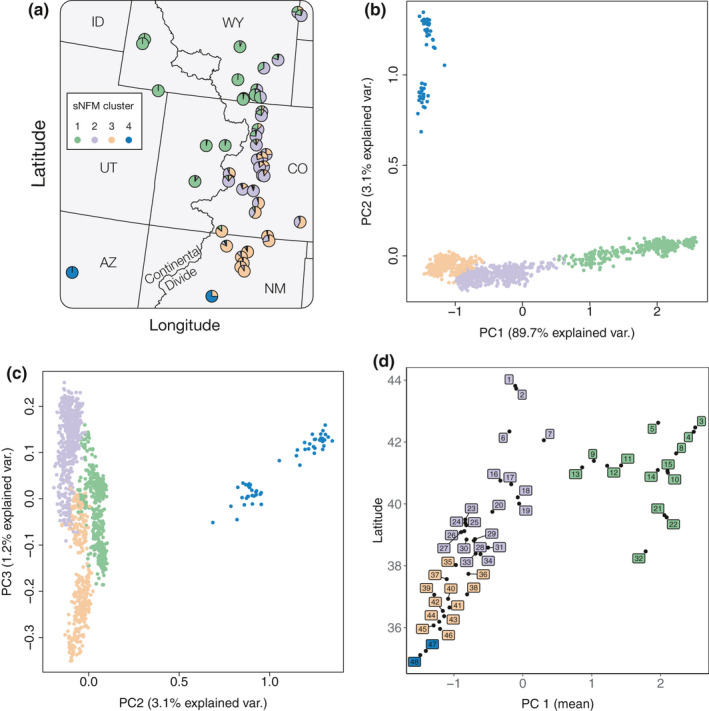
sNFM admixture and principal component analysis of*Cercocarpus montanus*. (a) Pie chart of sNFM admixture proportions from*k* = 4 ancestral gene pools (FigureS3) for each of the 48 populations collected in our study. (b) PC axes 1 and 2 and (c) 2 and 3 show continuous genetic variation across individuals within clusters. (d) Scatter plot of the mean PC axis 1 score for each of the 48 populations plotted with latitude to visualize geographic structure. Points are colored based on the predominant population assignment from admixture analysis

The first PC axis (PC1) accounted for 89.7% of the genetic variation among individuals of mountain mahogany and reflected latitude of origin and the effect of the Continental Divide as a barrier (Figure 2). PC2 accounted for 3.1% of genetic variation and separated two southwestern populations of *C. montanus* collected near Albuquerque, NM and Flagstaff, AZ. The first PC axis shows that mountain mahogany has continuous genetic variation in the southern portion of its range, and split into two separate clusters in northern latitudes (Figure 2 panel d).

The best supported number of clusters for sNFM admixture analysis was *K* = 4 (Figure S1). Populations were assigned to a single cluster based on the predominant population admixture coefficient of individuals within each population (Figure S2). The map of admixture composition shows that the genetic clusters were partitioned in geographic space (Figure 2, panel a), with more highly admixed zones between clusters. The genetic clusters occupied regions of the species environmental space with different multivariate centroids (Figure 4 panel a). Clusters 1 and 3 had no detected overlap in their environmental niche, while clusters 1 and 2 and 2 and 3 had partial, but not significant overlap in environmental space (Table S3).

**FIGURE 3:**
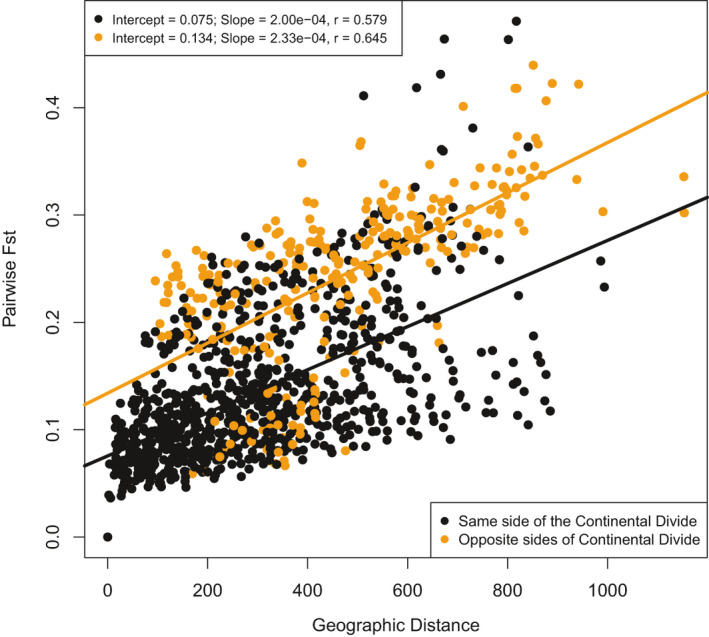
Matrix regression of pairwise genetic and geographic distances. Orange points are population pairs from opposite sides of the Continental Divide, while black points are from the same side of the Continental Divide. Two separate linear models results are listed and model line and summaries correspond to point colors

The mean Nei's *D*_A_ genetic distance between populations was 0.0346 (*SD* = 0.017), with a range of 0.009–0.108. Pairwise *F*_ST_ had an overall mean of 0.161 and a *SD* of 0.0856 (Figure S3). The mean *F*_ST_ among pairs of populations from opposite sides of the Continental Divide was 0.241 (*SD* = 0.079), while the mean FST among populations on the same side of the divide was 0.135 (*SD* = 0.07). Pairwise FST was positively correlated with spatial distance, and population pairs from opposite sides of the Continental Divide had elevated *F*_ST_ resulting from the effect of the topographic barrier (Figure 3). Growing season precipitation (GSP) and degree days less than 0°C (DD0) had the highest correlation with genetic differentiation, and were standardized and combined as a single mean Euclidean distance for each population pair to serve as the environmental predictor variable. The Bayesian linear model with the lowest DIC included both spatial and environmental distance as predictors of genetic differentiation (Table 1). The best predictor in a univariate model of genetic differentiation was geographic distance, followed by environmental distance, while the binary design matrix representing the Continental Divide was the worst predictor.

**TABLE 1:** Bayesian linear regression models and coefficients

Model	*β*_1_ (*SD*)	*β*_2_ (*SD*)	*μ* (*SD*)	DIC
GenDist ~ Environment + Geography	0.173 (0.013)	0.297 (0.0126)	−0.0028 (0.0457)	601.242
GenDist ~ Barrier + Geography	0.364 (0.0367)	0.301 (0.0132)	−9.63e−05 (0.0419)	679.119
GenDist ~ Barrier + Environment	0.465 (0.0408)	0.207 (0.0151)	0.000258 (0.0538)	921.633
GenDist ~ Geography	0.327 (0.0135)		−0.00132 (0.0494)	759.724
GenDist ~ Environment	0.229 (0.0156)		0.00428 (0.061)	1,026.883
GenDist ~ Barrier	0.533 (0.0435)		0.000125 (0.0491)	1,105.838

Note

Predictor variables were standardized using a z‐score prior to modeling. Genetic distance (GenDist) was calculated as Nei's *D*_A_. Environmental distance is a multivariate distance matrix of degree days less than zero and growing season precipitation. Geography is a pairwise geographic distance matrix. The smallest DIC indicates the best model.

The BEDASSLE analysis calculated the ratio of environmental and spatial distance effect sizes on genetic differentiation (α_E_: α_D_). We used growing season precipitation and degree days less than 0°C separately as environmental variables, as well as a binary design matrix representing the Continental Divide to quantify the effect of topography on genetic distance. A difference of one degree days less than 0°C was comparable to approximately 8 km, and a 1 cm change in growing season precipitation had the same effect on genetic differentiation as approximately 70 km geographic distance. The Continental Divide had the largest effect on genetic differentiation relative to spatial distance. Crossing the Continental Divide had the same effect on genetic differentiation in mountain mahogany as moving 1.7 × 107 km, a larger distance than our collection area.

**FIGURE 4:**
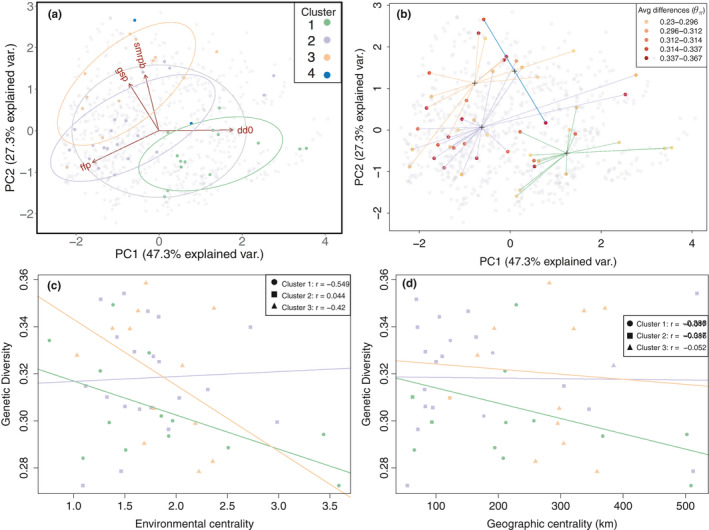
Principal component analysis of growing season precipitation (GSP), summer precipitation balance (smrpb), frost‐free period (ffp) and degree days below zero Celsius (dd0). (a) The genetic cluster assignment in environmental PCA space for each genetic cluster, as well as background occurrence points in gray. (b) Genetic diversity for each population (point) and the distance (lines) of each population to the cluster‐specific environmental centroid (crosses). Finally, genetic diversity (*θ_π_*) plotted over (c) environmental centrality and (d) geographic centrality. More central populations are closer to zero. Regression lines were modeled for each genetic cluster separately

We detected significant variation in genetic diversity across populations of mountain mahogany. Nucleotide diversity estimates were highly correlated (*r* > .9, *θ_π_* and *θ_W_*), and we therefore arbitrarily chose *θ_π_* for further modeling (Table S2). We analyzed genetic diversity with latitude, as well as the species' environmental and geographic centrality. We also checked the association between geographic centrality and the probability of occurrence score taken from a previous study (Crow et al., 2018) to determine whether the environmental niche was associated with geographic centrality. We found that genetic diversity was not associated with latitude (*p* = .266, *df* = 43, *R* ^2^ = 0.028). We checked the univariate correlation between genetic diversity and all climate and elevation data (Table S1), and selected GSP and DD0, as well as length of frost‐free period (FFP) and summer precipitation balance (SMPRB), as these were highly correlated with genetic diversity and had low collinearity. We combined these 4 climate variables to represent the multidimensional environment occupied by mountain mahogany. Genetic diversity was lower in populations farther from the species' multidimensional environmental centroid. Spatial centrality, however, was a poor predictor of *θ_π_*. The environmental distance to the centroid of each genetic cluster best described genetic diversity and had a negative correlation (Table 2). We also found significant variation among genetic clusters for the effect of environmental and spatial distance, namely genetic variation within the northern and southern genetic clusters (clusters 1 and 3) both had a significant relationship to environmental marginality, whereas within the central genetic cluster (cluster 2) diversity was not correlated with environment (Figure 4). Lastly, we found that geographic centrality was not correlated with the species' probability of occurrence (Figure S4).

**TABLE 2:** Summary of linear regression models and model selection criterion for the effects of geographic and environmental centrality on genetic diversity

Model	*β*_1_ (CI)	*β*_2_ (CI)	*μ* (CI)	*R* ^2^	adj. *R* ^2^	AIC
*θ_π_* ~ Environment (Env)	−0.01** (−0.03–0.001)	NA	0.36 (0.32–0.40)	0.106	0.085	−206
*θ_π_* ~ Geography (Geo)	−0.01 (−0.01–0)	NA	0.31 (0.31–0.32)	0.033	0.010	−202
*θ_π_* ~ Env + Geo	−0.01 (−0.03–0)	0 (−0.01–0)	0.36 (0.32–0.40)	0.109	0.067	−204

* *p* < .05.

** *p* < .01.

## 4 DISCUSSION

Mountain mahogany is commonly used in restoration programs (Paschke et al., 2000, 2003), particularly because it hosts nitrogen‐fixing actinobacteria that allow establishment in nutrient‐poor soils (Klemmedson, 1979), and provides important overwintering forage for wildlife (Turley et al., 2003). Despite widespread occurrence in the Rocky Mountain West, no prior ecological genetics study has characterized genetic structure across mountain mahogany's range. We sequenced 1,440 individuals from six U.S. states in the Southern Rocky Mountains to learn the extent of genetic heterogeneity across the geographic range and the environments occupied by the species.

We found evidence that genetic structure of mountain mahogany was affected by spatial and environmental distance, as well as topographic barriers. The results provide preliminary data for seed sourcing guidelines for mountain mahogany. Genetic structure is important to consider for species management, especially in a restoration setting where hundreds or thousands of individual plants are transplanted to a new site (Hufford & Mazer, 2003). These results have range‐wide implications for mountain mahogany shrubland management and lay the groundwork for critical decision‐making under environmental change.

The Bayesian model with the best fit for describing genetic differentiation included both spatial and environmental distance. Results from the BEDASSLE model, designed to disentangle the effects of spatial and environmental distance, showed that growing season precipitation (GSP) and the number of degree days less than zero (DD0) were associated with genetic structure in this species. The association between the environment and genetic differentiation supports seed sourcing guidelines that select collection areas that match the environment of the restoration site.

The Continental Divide was associated with higher genetic differentiation between Mountain mahogany populations, especially in central Colorado, where the Continental Divide is at high altitudes. Several studies have shown that the Continental Divide is a strong barrier to gene flow (Machado et al., 2018; Schield et al., 2018). However, to date, no published study has documented this in plant species. Several studies have found significant effects of topographic barriers on genetic differentiation in plant species, including seas (Jaros et al., 2017), lakes and terrain (Ju et al., 2018), rivers (Geng et al., 2015), mountains (Reeves & Richards, 2014; Zhu et al., 2017), and basins (Bontrager & Angert, 2018). Our data agree with these studies and indicate that populations from opposite sides of the Continental Divide are genetically more isolated, despite spatial proximity (Figure 3). Populations from the western slopes of the Rocky Mountains had high among‐population genetic differentiation, especially populations 3 and 4 (Figure 1 panel b and c). Populations 3 and 4 may have been founded separately from other western slope populations or may contain hybrids with a closely related species, *Cercocarpus ledifolius*, that co‐occurs in this region (Stutz, 1988). The two most genetically differentiated populations (47 and 48), in New Mexico and Arizona, respectively (Figure 1 panel b and c), inhabit isolated locations surrounded by desert regions with low habitat suitability (Crow et al., 2018). Populations 47 and 48 occur in relatively high temperature and low precipitation conditions, and may warrant further investigation.

Despite the heterogeneity of climatic conditions in our study area, we found that the best supported genetic clusters corresponded to populations in cohesive geographic regions (Figure 2). Further, the genetic clusters were associated with significantly different environmental space (Figure 4 panel a), which corroborates linear modeling results showing that spatial distance and environment are both factors related to genetic variation. Given these results, we analyzed patterns of genetic diversity across both spatial and environmental gradients for all populations, and for populations within each genetic cluster separately.

Model outcomes suggested that environmental centrality was a better predictor of genetic diversity than spatial distance. This analysis was completed for all sampled populations, as well as for individual genetic clusters. In both cases, genetic diversity was lower near the environmental niche periphery and not strongly correlated with geographic centrality. A previous study by Lee‐Yaw et al. (2017) found similar results, where genetic diversity of *Arabidopsis lyrata* ssp. *lyrata* was lower at the edge of the environmental niche, but not the limits of the sampled geographic range. Several meta‐analyses have shown that the geographic and environmental range limits do not necessarily coincide and that the geographic range frequently does not explain patterns of genetic variation (Eckert et al., 2008; Pironon et al., 2017). Another review by Lira‐Noriega and Manthey (2014) found that only about half of species ranges have any correlation between geographic and environmental marginality and that environmental marginality was consistently associated with genetic diversity, while geographic marginality was not.

Reduced genetic variation associated with environmental range limits does not distinguish whether populations occurring at range limits are demographic sinks maintained by immigration from more central habitat, or are important genetic resources adapted to marginal conditions by natural selection. However, the correlation of genetic diversity and environmental centrality bolsters our findings that genetic structure of mountain mahogany covaries with the environment. The lack of genetic homogeneity in mountain mahogany indicates that populations are not equivalent and caution should be taken when planning transfer of plant propagules, particularly during restoration.

Other studies of genetic variation near range limits have found contrasting results, even among populations within species. For example, Hargreaves and Eckert (2018) found that a subset of *Rhinanthus minor* populations near the range margin had lower fitness, while others were locally adapted. Aguirre‐Liguori et al. (2017) found that genetic diversity was lower near the geographic range margin of teosinte, and candidate adaptive SNPs were positively correlated with distance to the environmental niche centroid, arguing that populations near the geographic range margins were isolated, while populations near the edges of the environmental niche were locally adapted. In *Picea sitchensis*, populations proximal to the range margin were found to be more likely to carry rare alleles (Gapare et al., 2005), and a second study of *P. sitchensis* determined that populations near the range limit were locally adapted (Mimura & Aitken, 2010). These studies illustrate that range margins can harbor both source and sink genetic pools within species and that making predictions about population fecundity near range margins is difficult.

The results of our study suggest that populations of mountain mahogany have genetic structure across its range that is correlated with differences in the environment. The effect of the Continental Divide on genetic structure was significant. This suggests that transferring populations across the Continental Divide would increase the likelihood of maladaptation and subsequent risks for outbreeding depression among progeny of local and introduced plants. Degree days less than zero and growing season precipitation were significantly related to population genetic structure and differences in genetic diversity. These two variables could delimit collection sites when transferring seed sources during restoration. Choosing a commercial seed source or collection location that is most environmentally similar to the restoration site may increase chances of introducing adapted genotypes (Hufford & Mazer, 2003). In the case of mountain mahogany, preliminary seed collection zones could be delineated by the four genetic clusters. This is a practical approach given that the four clusters represent large spatial regions for collection despite considerable altitudinal and microhabitat variation. Whether populations near range margins are important resources for conservation of mountain mahogany remains unclear. Plants are subjected to biotic and abiotic stressors that influence population dynamics (Franklin et al., 2016; Pagel & Schurr, 2012), seed predators (Louda, 1982), pollinators (Biesmeijer et al., 2006), and dispersers (Merow et al., 2011). Additional studies are needed to determine the adaptive value of mountain mahogany populations along range margins for ecological restoration, particularly in light of changing climate conditions.

## CONFLICT OF INTEREST

The authors have no conflicts of interests to declare.

## AUTHOR CONTRIBUTION

**Taylor M. Crow:** Conceptualization (equal); data curation (equal); formal analysis (equal); funding acquisition (equal); investigation (equal); methodology (equal); writing—original draft (equal). **C. Alex Buerkle:** Conceptualization (equal); data curation (equal); formal analysis (equal); methodology (equal); validation (equal); visualization (equal); writing—review and editing (equal). **Daniel E. Runcie:** Formal analysis (equal); validation (equal); writing—review and editing (equal). **Kristina M. Hufford:** Conceptualization (equal); data curation (equal); formal analysis (equal); funding acquisition (lead); investigation (equal); methodology (equal); project administration (lead); writing—original draft (supporting); writing—review and editing (equal).

## Supporting information

Supplementary Materials

## Data Availability

Genotype estimates, allele frequencies, and climate data input files are available at: (https://doi.org/10.25338/B83P7Z).
