# Supplementary material for: Implications of genetic heterogeneity for plant translocation during ecological restoration
Source: Ecol Evol. 2021 Jan 16;11(3):1100–10. doi: 10.1002/ece3.6978 (PMC7863393; doi:10.1002/ece3.6978)
Supplement: Supplementary file 1 — Supplementary Materials [file ECE3-11-1100-s001.pdf]

Supplemental Information for:

Implications of genetic heterogeneity for plant translocation during ecological restoration

Table of Contents:

Contents

1 Tables **2**
1.1 Table S1 2
1.2 Table S2 3
1.3 Table S3 4

2 Figures **4**
2.1 Figure S1 4
2.2 Figure S2 5
2.3 Figure S3 6
2.4 Figure S4 7

1 Tables

1.1 Table S1

Table 1: Complete list of climate rasters downloaded from Moscow Forestry Sciences Laboratory.

Raster layer	Description
D100	Julian date the sum of degree-days >5 degrees C reaches 100
DD0	Degree-days <0 degrees C (based on mean monthly temperature)
DD5	Degree-days >5 degrees C (based on mean monthly temperature)
FDAY	Julian date of the first freezing date of autumn
FFP	Length of the frost-free period (days)
GSDD5	Degree-days >5 degrees C accumulating within the frost-free period
GSP	Growing season precipitation, April to September
MAP	Mean annual precipitation
MATTENTHS	Mean annual temperature
MMAXTENTHS	Mean maximum temperature in the warmest month
MMINTENTHS	Mean minimum temperature in the coldest month
MMINDD0	Degree-days <0 degrees C (based on mean minimum monthly temperature)
MTCMTENTHS	Mean temperature in the coldest month
MTWMTENTHS	Mean temperature in the warmest month
SDAY	Julian date of the last freezing date of spring
SMRP	Summer precipitation: (Jul+Aug)
SMRPB	Summer precipitation balance: (Jul+Aug+Sep)/(Apr+May+Jun)
SMRSPRPB	Summer/Spring precipitation balance: (Jul+Aug)/(Apr+May)
SPRP	Spring precipitation: (Apr+May)
WINP	Winter precipitation: (Nov+Dec+Jan+Feb)
ADI	Annual dryness index, DD5/MAP
SMI	Summer dryness index, GSDD5/GSP
PRATIO	Ratio of summer precipitation to total precipitation, GSP/MAP
AHMI	annual heat:moisture index, ((MAT+15)/MAP)*10
DEM	Digital elevation model

1.2 Table S2

Table 2: Nucleotide diversity and neutrality statistics for populations of mountain mahogany. Populations are numbered by the latitude which they were collected, starting with number one as the most northerly location.

Population	Latitude	Longitude	Elevation (m)	Continental Divide	θ_W	θ_π
1	43.82	-104.11	1341.81	East	0.354	0.354
2	43.73	-103.95	1526.28	East	0.218	0.279
3	42.47	-110.27	2268.40	West	0.224	0.273
4	42.33	-110.33	2419.40	West	0.281	0.294
5	42.62	-106.35	1949.87	East	0.248	0.294
6	42.34	-104.75	1464.91	East	0.257	0.305
7	42.06	-105.29	1815.78	East	0.271	0.315
8	41.63	-106.29	2318.00	East	0.299	0.3
9	41.39	-105.49	2328.78	East	0.39	0.349
10	41.01	-109.42	1966.34	West	0.189	0.23
11	41.24	-105.51	2403.97	East	0.3	0.299
12	41.23	-105.54	2293.81	East	0.349	0.321
13	41.18	-105.32	2379.68	East	0.24	0.284
14	41.09	-105.98	2709.43	East	0.271	0.289
15	41.06	-106.00	2370.32	East	0.339	0.329
16	40.75	-105.20	1882.50	East	0.348	0.336
17	40.63	-105.20	1664.04	East	0.356	0.347
18	40.21	-105.29	1713.08	East	0.353	0.344
19	40.01	-105.34	1873.13	East	0.256	0.306
20	39.74	-105.29	1974.16	East	0.298	0.31
21	39.63	-106.51	2285.58	West	0.288	0.288
22	39.56	-107.25	2019.81	West	0.356	0.334
23	39.49	-104.87	1908.44	East	0.309	0.329
24	39.38	-105.03	2058.67	East	0.368	0.352
25	39.31	-105.20	1958.49	East	0.201	0.273
26	39.12	-104.93	2320.48	East	0.299	0.313
27	39.09	-105.08	2414.29	East	0.258	0.296
28	38.81	-106.23	2711.30	East	0.319	0.34
29	38.86	-104.94	2174.94	East	0.274	0.306
30	38.85	-104.87	1886.53	East	0.31	0.325
31	38.59	-106.22	2883.39	East	0.232	0.3
32	38.46	-107.52	2678.89	West	0.299	0.302
33	38.39	-105.65	1996.78	East	0.329	0.327
34	38.37	-105.23	1839.59	East	0.268	0.31
35	38.02	-104.99	2303.27	East	0.324	0.328
36	37.74	-105.08	2040.84	East	0.378	0.339
37	37.57	-103.33	1515.11	East	0.269	0.299
38	37.07	-106.28	2741.43	East	0.221	0.283
39	37.06	-104.52	2133.30	East	0.387	0.359
40	36.93	-104.46	2163.72	East	0.301	0.305
41	36.65	-106.04	2682.42	East	0.246	0.29
42	36.54	-105.22	2577.56	East	0.359	0.339
43	36.37	-105.44	2549.87	East	0.389	0.347
44	36.19	-105.35	2708.31	East	0.207	0.278
45	36.07	-105.42	2722.81	East	0.361	0.348
46	35.96	-105.28	2190.80	East	0.313	0.323
47	35.25	-111.64	2282.53	West	0.261	0.328
48	35.11	-106.38	2048.98	East	0.349	0.367

1.3 Table S3

Table 3: Niche overlap D statistic. No significant overlap was found between the niche occuppied by any of the three major genomic clusters in this study.

Zone comparison	D statistic	p-value
One vs. Two	0.0798	0.545
One vs. Three	0.301	0.336
Two vs. Three	0	1

2 Figures

2.1 Figure S1

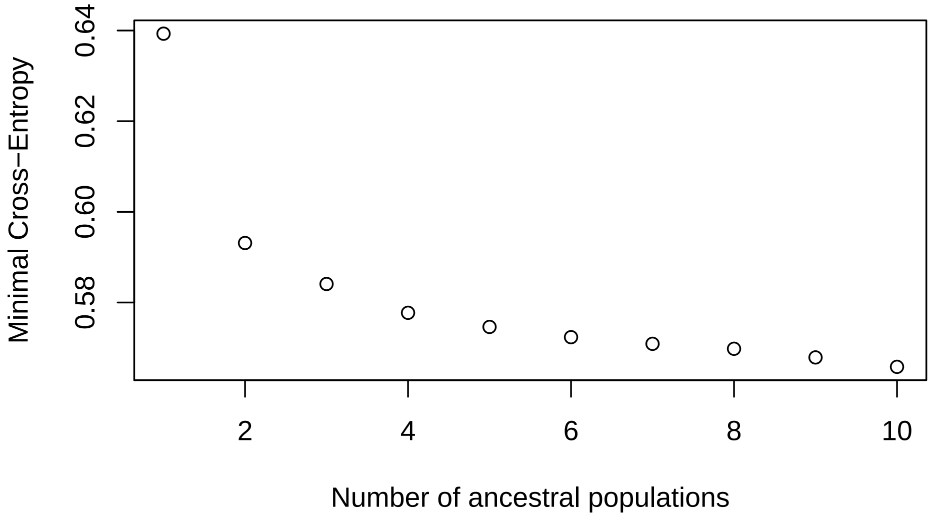

Figure 1: The minimum cross-entropy scores for K values 1-10.

2.2 Figure S2

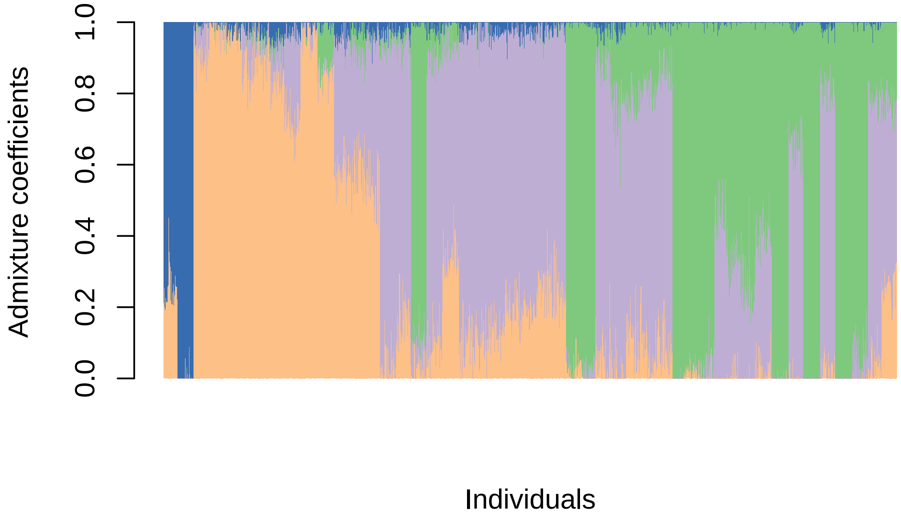

Figure 2: Barplot of sNFM admixture results for all 1309 individuals in this study. Samples are arranged by latitude.

2.3 Figure S3

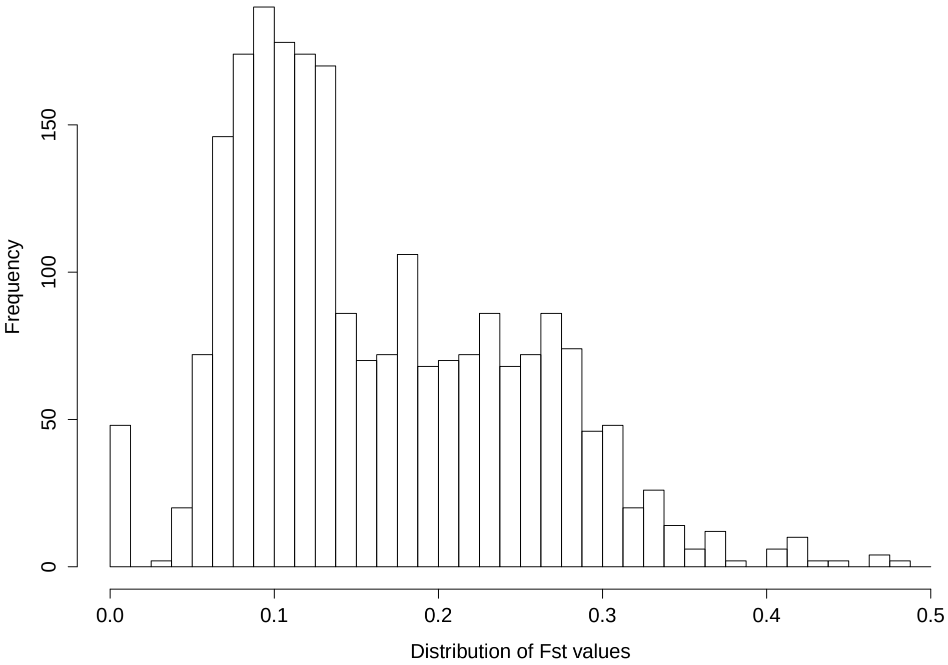

Figure 3: Distribution of F_{ST} values among populations of *Cercocarpus montanus* in our study. Notice that the data are skewed, likely due to the effect of isolation by barrier.

2.4 Figure S4

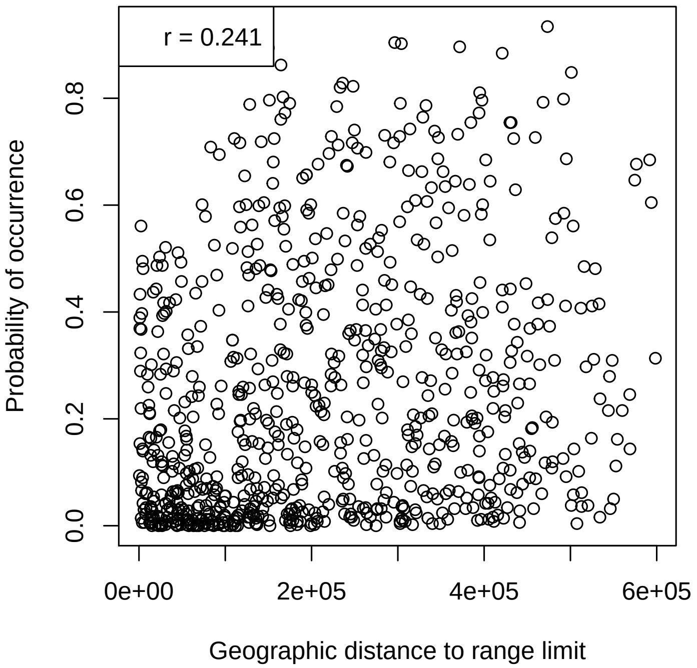

Figure 4: Randomly sampled one thousand points across the range of *Cercocarpus montanus* to determine the relationship between the probability of occurrence and the geographic distance to range limit. Very weak positive relationship indicates that in heterogeneous environments, the distance to the range margin does not have a strong relathionship with the quality of habitat.
